# Recent Developments and Challenges in the Treatment of Acute Leukemia and Myelodysplastic Syndromes: A Systematic Review

**DOI:** 10.7759/cureus.72599

**Published:** 2024-10-29

**Authors:** Nawal Rafiq, Muhammad H Khan, Mashaal Sahibzada, Shakeeb Ahmad Khan, Aswani Syamprabha Vijayan, Najeeb Ullah, Chenny Koodarath, Hira Khalil, Umar Azam Ali, Falaknaz Saleem, Sarah Almounjed, Ibrar Khaliq

**Affiliations:** 1 Accident and Emergency, Rehman Medical Institute, Peshawar, PAK; 2 Accident and Emergency, Health Department-KP, Peshawar, PAK; 3 Medical High Dependency Unit, Northwest Teaching Hospital, Peshawar, PAK; 4 Emergency Medicine, Lady Reading Hospital, Peshawar, PAK; 5 Internal Medicine, SK Hospital, Thiruvananthapuram, IND; 6 Internal Medicine, Rehman Medical Institute, Peshawar, PAK; 7 Critical Care Medicine, SK Hospital, Thiruvananthapuram, IND; 8 Internal Medicine, Ayub Medical College, Abbottabad, PAK; 9 Internal Medicine, George Eliot Hospital, Nuneaton, GBR; 10 General Surgery, Liv Hospital, Istanbul, TUR; 11 Internal Medicine, Services Hospital, Lahore, PAK

**Keywords:** acute leukemia, challenges, developments, economic effects, myelodysplastic syndrome, quality of life, survival rate, treatment

## Abstract

The diagnosis of acute leukemia (AL) and myelodysplastic syndrome (MDS) is critical due to their rapid progression and varied survival rates between children and older adults. These diseases are associated with significant mortality, highlighting the need for strategies to reduce the global burden of AL and MDS. Their direct involvement with the blood, bone marrow, and extramedullary sites complicates treatment management. However, recent therapeutic advancements offer hope for the long-term management of AL and MDS. This systematic review followed the guidelines put forth by Preferred Reporting Items for Systematic Reviews and Meta-Analyses (PRISMA) to assess recent developments and challenges in the treatment of AL (including acute myeloid leukemia (AML) and acute lymphoblastic leukemia (ALL)) and MDS. Databases such as PubMed, Google Scholar, NCBI, Scopus, Blood Journal, Cochrane Library, and Leukemia Gene Atlas (LGA) were used to retrieve articles published from 2017 to 2024, with the last search conducted in August 2024. A total of 12 peer-reviewed studies were selected based on specific inclusion and exclusion criteria. These studies reveal advancements in the diagnosis, classification, and treatment of AL and MDS, including long-term disease-free survival, complete remissions, and improved patient outcomes in those over 75 years of age. Less toxic treatment methods, such as targeted therapies, immunotherapies, and bispecific T-cell engagers, are particularly beneficial for older adults with ALL. Significant progress has also been made in understanding the genetic mutations in AML, leading to more personalized therapies. In MDS, a combination of chemotherapy, immunosuppressive treatments, targeted therapies, and stem cell transplants has shown high efficacy. However, challenges remain, including high initial treatment costs, limited patient access, inadequate awareness, insufficient employee training, and the lack of accurate treatment models. Despite these hurdles, these advances provide promising options for improving the quality of life for patients with AL and MDS.

## Introduction and background

Acute leukemia (AL) is among a rapidly progressing cancer type of bone marrow and blood resulting from an excessive number of immature white blood cells known as blasts [1]. It turns into two main types, including acute lymphoblastic leukemia (ALL) and acute myeloid leukemia (AML). However, ALL is a result of immature lymphoid cell proliferation, and AML is an outcome of myeloid cell proliferation in the blood. ALL diagnosis is common among children, and AML is found in any age group, but it is prevalent among adults [2]. According to the Leukemia and Lymphoma Society, in 2023, estimated cases of AML were 20,380 (34%) and of ALL were 6540 (11%) among adults and children in the United States [3]. The global burden of ALL and AML has been reduced in the United States after the implementation of the latest techniques, as the total cases of leukemia in 2019 were 437,337, and in 2023, these were reduced to 59,610 [3].

Some studies suggest that AML ascends from the somatically acquired mutations that are a highly progressive process with the age of human beings [4]. AML can also be raised from de novo and secondary in various other processes, like a precursor of hematological disorders and exposure to cytotoxic or immunosuppressive therapies. Furthermore, the median age in the United States for AML diagnosis is 68 years; however, the incidence rate increases with age [5]. The overall survival (OS) among patients below 65 years was improved from eight months (1975-1979) to 46 months (2010-2014) [6]. At the same time, the survival rate among <65-year-olds also increased. As per Yi et al., OS rates from AML among two-year-olds are 32% and among five-year-olds are 24% [7].

AML is a malignant proliferation and transformation of the lymphoid progenitor cells within the blood, bone marrow, and extramedullary sites [8]. Moreover, 80% of AML is prevalent among children; however, it is denoted as a devastating illness among 20% of adults [9,10]. The AML incidence rate has steadily increased globally, which indicates an increased requirement for more healthcare resources for addressing problems associated with age. AML is among the most common acute leukemia present among adults, accounting for 80% of cases among adults. It directly affects older adults, as the median age of diagnosis is 68 years or older [10-12].

Myelodysplastic syndrome (MDS) encompasses a group of diseases of the bone marrow and blood with varying degrees of life expectancy and severity [13]. MDS starts from the alterations and variations in the normal stem cells present in the marrow [14]. This results in a marrow filled with a huge number of developing blood cells [15]. On the other hand, MDS shows a blood deficiency in cell numbers due to cells destroyed in the marrow before their release into the bloodstream [16]. In a patient with MDS, blasts are usually constituting more than 5% of the marrow cells. However, in a person with AML, blasts become 20% of these cells. MDS is also known as "smoldering leukemia," but the terms can mislead the assessment as it indicates MDS is only serious if it evolves into AML. Furthermore, the most common MDS subtypes include refractory anemia, comprising 15.5% of blasts excess, and refractory cytopenia, counting multilineage dysplasia, which contains 8.3% of blasts [17]. Furthermore, patients with MDS diagnoses that are not particularly specified constitute 62.7% of the entire MDS cases. Such a prevalence is found among 58,835 people in the United States who are living with MDS [18].

The treatment for ALL was followed by tyrosine kinase inhibitors for intensive chemotherapy to improve the outcomes since 2000 [19-21]. The mid-20th century acted as a breakthrough in the development of cancer treatment due to the introduction of chemicals within the body as they assisted in the remission of patient lymphoma. Further development was of immunotherapy, precision medicine, and targeted therapies that have brought transformation in cancer treatment [21]. Moreover, the genomics-based advancement in the ALL treatment followed personalized treatment as per individual genetic makeup. Immunotherapies were developed to harness the immune system and assist in the fight against cancer cells [19,20]. Moreover, minimally invasive procedures, telemedicine, and robotic surgery were improving patient care.

Moreover, AML treatment has been found with substantial progress, starting from the "3+7 regimen" as a standard intensive chemotherapy approach applied to younger patients [22]. In this case, the survival rates were still ranging from 40% to 50% among young patients and 30% to 40% for older patients. On the other hand, the patients above 60-65 years were having poor outcomes with the regimen as it was related to the high mortality rate and a lower rate of long-term survival. Targeted therapies also act as novel treatment options, including BCL2 inhibitors, IDH inhibitors, and FLT3 inhibitors. These targeted therapies raised questions about the optimal standards of care of the "3+7 regimen" [22-24].

The therapeutic landscape has improved over time for MDS, and allogeneic hematopoietic stem cell transplantation (allo-HSCT) is among the potential curative options [25]. However, the issues are related to its accessibility, and a few patients are a great fit for the treatment due to its intensity. Such treatments for most patients are risk-adaptive and non-intensive. These approaches following cytopenias were aimed at improving the quality of life and delays in the progression of the disease [18,26]. Challenges related to the initial treatment options for ALL, AML, and MDS were major side effects, drug resistance, relapses, access to treatment, availability for patients, and care coordination. Furthermore, quality of life, costs of treatment, insurance coverage, and clinical trials are also among the major issues related to the ALL, AML, and MDS. Despite the presence of the evolution of the treatments for ALL, AML, and MDS, there are some aspects that need to be explored regarding the latest technology treatment options [27].

The primary focus of the current systematic review is to address the issues linked with treatment and its impact on various factors related to patients. A lack of information and fewer studies explaining the treatments with various challenges for ALL, AML, and MDS indicate the need for a comprehensive study that can review the treatments and challenges associated with the latest technology products regarding quality of life, span of treatment, impact on the repression, side effects, stability of patient, and other psychological factors. The review will impact the limited understanding of the latest technologies and therapies developed for the ALL, AML, and MDS in terms of treatment effectiveness, economic factors, psychological factors, and personal factors by limiting the bias in current understandings. The in-depth analysis will help to provide a precise understanding of the latest treatments that are useful for children, adults, and older adults.

The rationale for conducting the current systematic review is to increase knowledge about ALL, AML, and MDS treatment and challenges. This will help to explore the ideal situations that can be adopted, along with their pros and cons. The advancement in technological instrument and their economic impact will assist the stakeholders in understanding the costs related to the treatment options. Furthermore, the review will also help to narrate how the perspective can further be applied regarding psychological issues and quality of life after such treatment options for ALL, AML, and MDS. The purpose is to explore the needs of patients in different age groups, and it will highlight the required policy options that can assist in reducing the challenges for patients in order to access treatment for ALL, AML, and MDS.

## Review

Materials and methods

The current systematic review assesses recent developments and challenges in the treatment of AL (ALL and AML) and MDS. To ensure a comprehensive and unbiased assessment, the review followed the Preferred Reporting Items for Systematic Reviews and Meta-Analyses (PRISMA) guidelines. We searched multiple databases, including PubMed, Google Scholar, NCBI, Scopus, Blood Journal, Cochrane Library, and Leukemia Gene Atlas (LGA), to gather relevant literature. The search was conducted for articles published between 2017 and 2024, with the last search conducted in August 2024. No ethical registration was necessary, as this study involved the analysis of pre-existing published data and did not directly involve human subjects.

Eligibility Criteria

The eligibility criteria indicate studies that are included based on some important conditions that are presented in Table 1 which shows the inclusion and exclusion criteria. There can be observed the criteria established with a clear explanation of what is included in the current study and what is avoided to make the study accurate and reliable.

**Table 1:** Inclusion and exclusion criteria. AL: acute leukemia; MDS: myelodysplastic syndromes; RCT: randomized controlled trial

Criteria	Inclusion	Exclusion
Timeline of publications	Research papers published from 2017 to 2024	Research papers published before 2017
Type of study selected	Systematic reviews, meta-analyses, RCTs, longitudinal studies	News websites, editorials, opinions, non-peer-reviewed articles, and case reports
Subject factors	Studies focusing on the treatment developments and challenges in AL (AML and ALL) and MDS	Studies not addressing the treatment developments or challenges specific to AL (AML and ALL) or MDS
Language	Research works published in English only	Research published in languages other than English
Availability of data	Full-text research articles with clear and complete data accessible online	Studies with only abstract availability, incomplete data, or restricted access
Methods	Studies that explore AL and MDS treatment developments, diagnostic tools, and associated challenges	Studies that focus only on pathology and prevalence or offer superficial overviews of treatment
Measure of research outcomes	Studies that report treatment benefits and challenges for AL and MDS patients, with a focus on quality of life	Studies with unclear measures of outcomes or that fail to adequately address quality-of-life impact

Search Strategy

The search strategy was developed by selecting key phrases and keywords based on the research objectives. Careful consideration was given to ensure that relevant data were captured. Boolean operators were employed to manage precise keyword combinations. The search terms used were related to the treatment developments and challenges in ALL, AML, and MDS. Keywords included "Acute Lymphoblastic Leukemia treatment", "Acute Myeloid Leukemia challenges", "MDS treatment advancements", "immunotherapy in ALL", "targeted therapies for AML", "stem cell transplantation for MDS", "quality of life in leukemia", and "chemotherapy in AML and ALL".

The Boolean operators "AND", "OR", and "NOT" were used to refine the search further. For example, searches like "ALL AND immunotherapy", "AML AND targeted therapies", and "MDS AND stem cell transplantation" were applied to access the most relevant studies. The goal was to focus on studies that specifically addressed treatment developments and challenges in ALL, AML, and MDS, as well as their impact on patient outcomes.

Data Extraction

The data extraction process was conducted independently by two researchers from our team (names can be specified if required). This ensured fairness and minimized potential biases during the screening process. The procedure included an initial review of abstracts and titles and the removal of duplicates, as well as filtering based on language (English only), full-text availability, and timeline (2017-2024). Following the initial screening, a comprehensive evaluation of the full-text articles was conducted.

Keywords were applied during the screening process to help eliminate irrelevant studies, and inclusion/exclusion criteria were strictly followed to maintain study relevance. The extracted data were organized and managed using Rayyan QCRI (Rayyan Systems Inc., Cambridge, Massachusetts, United States), a widely used tool for systematic reviews. This software helped in streamlining the selection process and ensuring consistency. The systematic review table included the author's name, publication year, treatment area (ALL, AML, or MDS), study design, findings, and limitations.

The studies were further evaluated using the Critical Appraisal Skills Programme (CASP) tool to assess their methodological rigor, validity, and reliability. This quality assessment helped in identifying potential biases and limitations. The results were then categorized based on research areas of interest, methods used, and conclusions drawn. Finally, ethical considerations were adhered to by properly referencing original authors and ensuring no alterations were made to the meaning of outcomes in prior studies.

Data Analysis and Synthesis

The data collected following the inclusion and exclusion criteria and PRISMA flowchart were synthesized in the form of a chart. It includes the narrative text method to explore the treatment development and challenges of ALL, AML, and MDS.

Results

Study Selection Process

**Figure 1:**
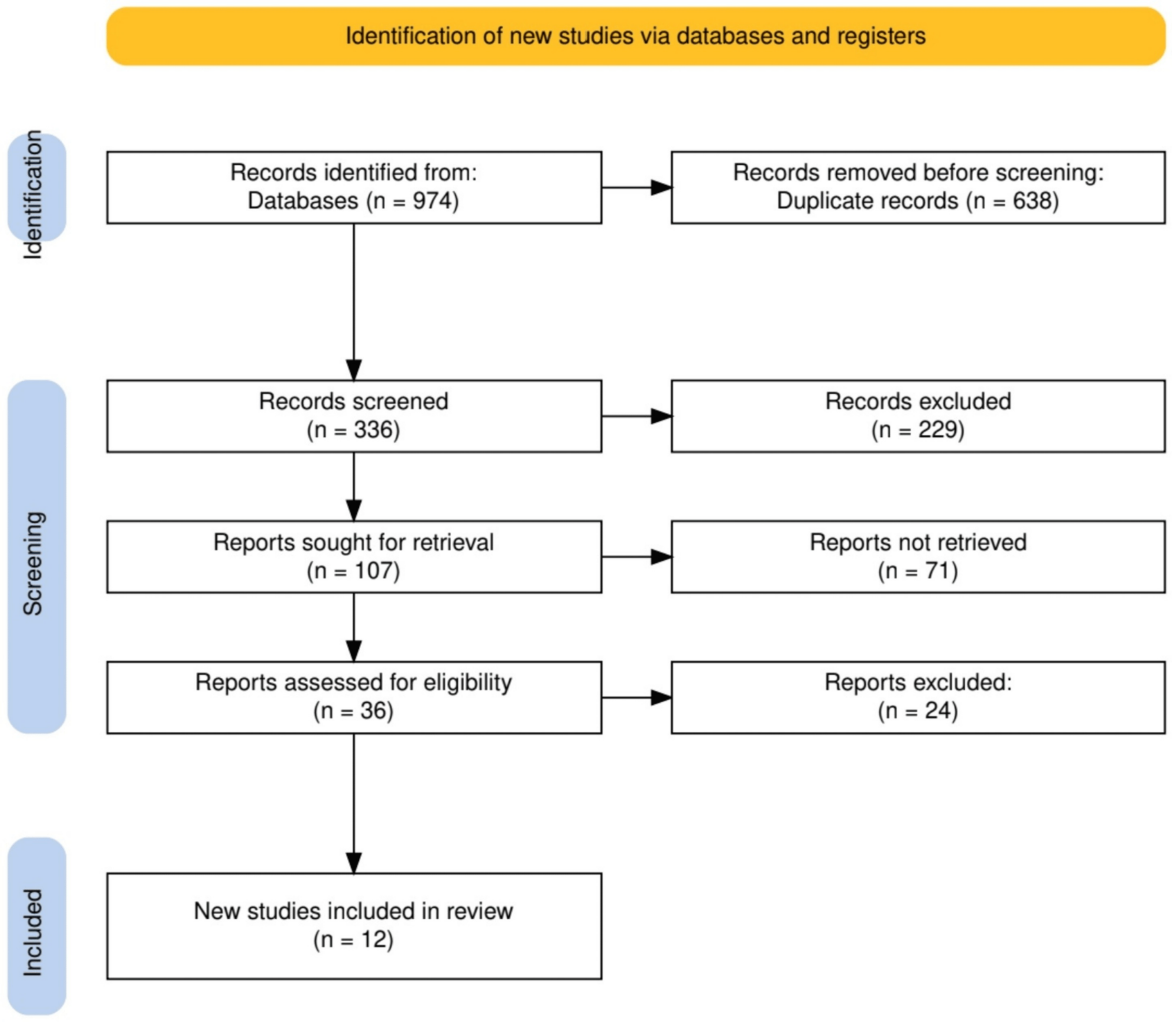
The PRISMA flowchart represents the study selection process. PRISMA: Preferred Reporting Items for Systematic Reviews and Meta-Analyses

The study selection process began with the identification of 974 records from various databases. After removing 638 duplicate records based on the selected timeline, 336 records were screened. Of these, 229 records were excluded for not meeting the abstract similarity criteria. The remaining 107 reports were sought for retrieval, but 71 were not retrieved due to access issues. Subsequently, 36 reports were assessed for eligibility, with 24 being excluded based on reliability criteria. Ultimately, 12 studies were included in the final review, comprising both qualitative and quantitative analyses. The study selection process is elaborated in Figure 1.

Systematic Review of Literature

The studies selected for the current review are presented in Table 2. All articles and research reviews in the current study followed high-quality and selected content related to the objectives of the current study.

**Table 2:** Systematic review of recent developments and challenges in the treatment of MDS. AML: acute myeloid leukemia; AZA: azacitidine; VEN: venetoclax; FLT3: FMS-like tyrosine kinase 3; ALL: acute lymphoblastic leukemia; CAR: chimeric antigen receptor; MDS: myelodysplastic syndromes; CPX-351: a liposomal formulation of daunorubicin and cytarabine; DAC: decitabine; B-ALL: B-lineage acute lymphoblastic leukemia

Reference	Year	Study design and sample size	Treatment development or challenges	Findings	Impact on patients
Dozzo et al. [28]	2023	Qualitative, systematic review	The novel liposomal formulation techniques to cure AML are beneficial. However, the complicated production process increases initial costs and improves outcomes	Chemotherapy takes two phases and comes with a harsh impact on patients >75 years. The novel liposomal formulation is a 7+3 regimen	Increased remission rate, drug sustain in the blood and bone marrow, survival rate, and hematological recovery from 60 to 75 years
Abaza et al. [29]	2024	Quantitative, randomized control trial and 765 patient sample	Allogeneic stem cell transplantation. In secondary AML, the subgroup analysis indicated AZA/VEN has survival benefits among elderly patients	Eleven new agents were assessed after 2017 to treat AML, and AML clinical treatments are possible with the help of targeted therapeutic regimens	Improved patient outcomes with FLT3 mutations. An overall survival rate was also increased among elderly patients
Gómez-De León et al. [30]	2023	Qualitative literature review	Lack of access to the latest development, care capacity, and novel agents	The current treatment technologies are better options available for patients, but a significant number of patients are not able to access these treatments	Mortality is higher in the United States as compared to developing countries
Bhansali et al. [31]	2023	Qualitative based on literature	Hypomethylating agents, AZA, and VEN are moving up with the ranks. It has different treatment levels among people unfit for AML-intensive chemotherapy	AML is of a complicated nature, and for this, the treatment options are going to be based on various active, low-intensity therapies. Molecular-informed data can effectively assess the subtypes of AML	Due to segregated treatment for different age groups and patients with various mutations, it becomes easy to assess the subtypes through molecular-informed data
Li et al. [32]	2020	Qualitative, literature review based	The treatment development is antibody-based immunotherapies	There has been an 81% remission rate through these therapies, which are also less toxic. The median age is 7.7 months of survival after immunotherapies	Immunotherapies leave a positive impact on patient health as it is also useful among elderly patients as compared to chemotherapies
Ekpa et al. [33]	2023	Qualitative, systematic review 2012-2023	Generic and environmental factors are barriers, and increasing the incidence rate of ALL, financial cost is another significant challenge	There has been an improvement in the survival rate in the last five years; however, the incurable disease rate is still high	ALL treatments over the decade have been improved with the latest technology development
Aureli et al. [34]	2023	Qualitative, literature based	Hematopoietic stem cell transplantation and chemotherapy have unsatisfactory cure rates	The recent development for ALL in the form of bispecific antibodies, CD19 CAR T-cell therapy, and B-ALL management added a positive impact among ALL patients	Developments are effective for quality of life, early cure, and least severe events as compared to chemotherapy and hematopoietic stem cell transplantation
Lv et al. [35]	2022	Qualitative literature review	Immunotherapies are useful to increase immune response among children. However, toxicity and resistance related to immunotherapies are among the major challenges	Immunotherapy development has increased the long-term survival rates among children as these are helpful in increasing immune response, cure rate, and quality of life among patients	Patient outcomes are increased with high immune response, but toxicity is still present among such treatments
Niscola et al. [36]	2024	Qualitative systematic review	Molecular data-based prognosis. Novel drugs have a high tolerance rate for patients and have safe formulations like CPX-351 and luspatercept	The treatment option has been expanded currently for MDS with novel therapies that are treating anemia with accuracy and safety with oral combinations	Novel targeted drugs are positively impacting patient outcomes and shifting hematological practice
Platzbecker et al. [37]	2021	Qualitative literature review	Patients have low- to high-risk MDS; for high-risk MDS, the treatment options are changed, and current treatments are not accurate for high-risk MDS	The rate of relapse is high among high-risk MDS patients, and existing approved therapies are not eligible for low-risk patients. Therapies need to be developed as per patient needs	Patients at low risk received treatment, and their quality of life improved, but the patients with high risks were not able to get curative treatment
Sekeres et al. [38]	2023	Qualitative literature review	Most clinical trials are conducted in huge settings, but patients with MDS in the United States are treated in community settings	Clinical trials need to reflect disease evolution and the patients with advanced MDS and treat these patients in the real world	Improved quality of life, anemic patient response criteria, improvement in the symptoms
Stanchina et al. [39]	2021	Qualitative, critical literature review	Therapeutic discoveries for MDS are limited: correction of cytopenia and symptomatic management, limited in vitro models, lacking a specific environment for experiments	Currently, only five approved drugs are present to treat MDS, including luspatercept, AZA, lenalidomide for 5q deletions, oral DAC-cedazuridine, and DAC	The challenges are limiting treatment options for MDS patients and the accuracy of the treatment model

Discussion

AML incidences increase with age, and these are related to poor prognostic outcomes. Age is among the essential factors for dictating the treatment options and prognosis [28]. For the treatment of AML, the disease subtype, the overall status of the patient, and the personal preferences of patients to achieve quality of life are the main considerations. The novel liposomal formulation follows a 7+3 regimen with a combination of two delivery drugs daunorubicin to cytarabine and a molar ratio of 1:5. This gives a synergic outcome for sustaining drugs in the bone marrow. The development is effective due to improved remission rates, high survival, less toxicity, improved quality of life, and issues related to initial costs that are because of the complicated production process of drugs.

AML treatment has evolved rapidly after 2017, as before this the treatment options were anthracycline-based induction and standard cytarabine chemotherapy. However, single-agent hypomethylating agents, such as azacitidine, have been shown to be beneficial for patients over the age of 75 with AML, offering a less intensive therapeutic option [29]. The treatment evolution was followed by the introduction of various new agents. However, secondary AML was an area of concern even after such interventions due to poor response and unmet needs. Furthermore, a combination of targeted therapeutic regimens for IDH1/2 or FLT3 mutation patients came up with great clinical dealing for secondary AML as it was a one-size-fits-all for all [29]. Even in the presence of such a therapeutic arsenal, outcomes were also improved for AML arising from MDS. It tends to reduce the secondary adverse effects, and the response is optimized.

The AML diagnosis is challenging, considering the need for specialized diagnostic tools like flow cytometry, molecular assessment, and cytogenetics that further require the treatment through proper classification, risk stratification, and implementation of targeted therapies [30]. Furthermore, the main challenges are related to the lack of education, availability, training, and coverage for such expensive tests. The cost of treatment also increased for the novel methods. Such aspects raise the question about therapy that should be recommended to patients as per the current standards. For example, in Latin America, most likely get AML [30]. This led to chemotherapy shortages because of poor governmental administration and leadership, which is also faced in various countries. The results are complications for patient management when applying the substitute and creative drugs that are available, but this negatively impacts the outcomes.

Advanced AML treatment has rapidly increased in the last 10 years as molecular profiling came in the form of advanced methods that have increased the understanding of the pathobiology of disease and its therapeutic vulnerabilities [31]. This has indicated the subtypes of AML due to the molecular and genetic basis showing the recent updates from the World Health Organization. Armamentarium was found among the latest drugs as approved and rapidly expanded for AML treatment. However, another aspect of the low-intensity induction therapy with venetoclax and hypomethylating agents is to improve the outcomes among those with poor prognosis [31]. Molecular-informed data can help to reduce the complex diagnosis and further treatment of MAL among various age groups.

Recent immunotherapies are developed for ALL through T cells redirecting antibodies, naked antibodies, and antibodies linked with cytotoxic agents [32]. When compared with chemotherapy, the suggestion over the antibody-drug conjugates (ADC) are relapsed, and a complete remission is observed with ALL as a rate of 81% is found with 7.7 months survival and less toxicity. The use of blinatumomab also comes with an 80% remission rate and 7.7-month survival [32]. The antibody-based therapy is coming with a great response with ALL. The safety and efficacy increased among elderly patients with these antibodies.

Ekpa et al. [33] described ALL with various conditions in the systematic review conducted over a 10-year period from 2013 to 2023. The treatment developments were found for ALL in Down's syndrome, T-lineage acute lymphoblastic leukemia (T-ALL), and B-lineage acute lymphoblastic leukemia (B-ALL). There has been assessed almost 20% more risk found for ALL among Down's syndrome. The genetic risk factors are related to the environment and genetics. The diagnostic challenges are related to the genomic analysis of biomarkers [33]. These tend to define the targeted therapies and their importance that are less potent. A five-year survival rate was found within the United States and Canada among ALL patients due to improvements in technology, diagnosis, drugs, awareness, and follow-up modalities. However, the issue is similar for the patients who access such treatments.

ALL survival rate in children is high as compared to adults as it is poor among older people >60 years [34]. Promising development has been found in the ALL over the years, including ADC, bispecific antibodies, and chimeric antigen receptor (CAR)-based therapies. These developments are beneficial for children and adults at the same time. Chemotherapy is related to adverse events after a long term. Low socioeconomic countries are not able to get these treatments, indicating the challenges of treatment and equal opportunity for patients to improve their quality of life.

In another study [35], the new developments are discussed for ALL through immunotherapies. More capable therapies are CAR T cells and bispecific T-cell engagers that are capable of increasing immune response among very young ALL patients. Lv et al. [35] explained the developments among pediatric ALL using refractory or relapsed B-cell ALL that improve the cure rate and quality of life among patients but need market approval. With such benefits, the organization is also facing challenges related to toxicity and resistance. For example, blinatumomab is approved in the market for treatment, but issues related to such treatments include short-shelf life due to its low molecular weight, limits to wider clinical application, and continuous intravenous infusions. 

Niscola et al. [36] explained that a significant advancement has been observed in the recent decade to treat MDS through updated classification through genomic data, which increased the risk of stratification as it is leading toward personalized treatment approaches with identified therapeutic targets. On the other hand, practically, the introduction of molecular data has turned patients into the higher-risk categories of MDS. The new classification system for MDS has led to the development of innovative and well-tolerated drugs, such as CPX-351, which is particularly beneficial for treating high-risk MDS (HR-MDS) and shares efficacy with therapies for AML [36]. New drug disparities of availability within all continents indicate a challenge for equal treatment for MDS patients.

Current treatments of MDS are not curative as these lead to an impact on the quality of life negatively, and patients observe relapse to the treatment in the first session [37]. This shows a particular aspect of the unmet needs of patients for the latest treatments and effective strategies for managing MDS. The current advancements have increased MDS-based understanding regarding its pathogenesis. This indicates the diverse nature of the genetic abnormalities faced by MDS patients that further need a complicated and personalized treatment option. The incidence rate of MDS increases progressively from young age to elderly as it is highly progressive among >80-year-old individuals [37].

The clinical trial design for MDS faces various challenges in facilitating the drug design and meeting the patient's needs effectively [38]. Such challenges are cumbersome response criteria, validation of outcomes reported by patients, and standardized transfusion threshold. Clinical trials also need to be based on the advancing age of the patients with MDS, as they can be treated in real-world settings to increase the likelihood of active drugs [38]. However, for patients having low-risk MDS and for anemic patients, the response criteria should be symptom improvements, transfusion dependency, and quality of life. High-risk patients need to be involved in the guidance for dosage reduction and prevention that can limit the specification and efficacy of the dosage delays and OS response.

As per the study by Stanchina et al. [39], targeted therapies in HR-MDS development are developed as pevonedistat is one of the inhibitors with great benefits used in combination with azacitidine (AZA), and the treatment paradigm can be altered in this case for HR-MDS. At the same time, venetoclax in AZA combination is assessed as a first-line setting. In phase 3 trials are immunoglobulins and immunotherapies that help to restore the immune function towards normal working.

Despite the comprehensive nature of this systematic review, several limitations must be acknowledged. First, the review only included studies published in English, potentially excluding relevant research in other languages. Additionally, the analysis focused on studies published within the last five years (2017-2024), which, while ensuring a focus on recent developments, may have overlooked earlier significant findings. Furthermore, the reliance on published literature and publicly accessible data may have led to publication bias, as studies with negative or inconclusive results are less likely to be published. Lastly, while systematic reviews and meta-analyses were included to provide a broader scope, this may have introduced redundancy, as some findings were repeatedly referenced across multiple studies. Future research could benefit from including non-English studies, older studies, and unpublished data to provide a more comprehensive perspective.

## Conclusions

The study aimed to assess the developments and treatment challenges of AL (AML and ALL) and MDS. The systematic review indicated the prognosis of disease among children and adults, but MDS is diagnosed among older individuals. The current treatment and development of ALL, AML, and MDS indicate the advanced immunotherapies and a mixture of treatment with various regimens and antibodies that are progressively acting to improve the quality of among ALL, AML, and MDS patients. The main challenge related to the latest developments is the access of individuals as most of these treatments are only served within sophisticated setups of developed countries that indicate the unmet needs of the developing countries with ALL, AML, and MDS. The developments are also beneficial in terms of events and toxicity linked with chemotherapy options. Immunotherapies and antibody-based developments are coming with more safety and a high progress rate. It led to a reduction in toxicity for patients, but the cost issues are similar for entire developments in the AML, ALL, and MDS due to the production complexities of such materials.
